# Anti-inflammatory activities of Qingfei oral liquid and its influence on respiratory microbiota in mice with ovalbumin-induced asthma

**DOI:** 10.3389/fphar.2022.911667

**Published:** 2022-08-23

**Authors:** Jun Zheng, Qian Wu, Liang Zhang, Ya Zou, Meifen Wang, Li He, Sheng Guo

**Affiliations:** ^1^ Department of Traditional Chinese Medicine, Shanghai Children’s Hospital, School of Medicine, Shanghai Jiao Tong University, Shanghai, China; ^2^ Department of Emergency Medicine, Putuo Hospital, Shanghai University of Traditional Medicine, Shanghai, China; ^3^ Department of Pediatrics, Sanmen People’s Hospital, Taizhou, Zhejiang, China; ^4^ Department of Endocrine, Genetics and Metabolism, Shanghai Children’s Hospital, School of Medicine, Shanghai Jiao Tong University, Shanghai, China

**Keywords:** airway inflammation, asthma, metagenomic functional prediction, Qingfei oral liquid, respiratory microbiota

## Abstract

Dysbiosis of respiratory microbiota is closely related to the pathophysiological processes of asthma, including airway inflammation. Previous studies have shown that Qingfei oral liquid (QF) can alleviate airway inflammation and airway hyper-responsiveness in respiratory syncytial virus-infected asthmatic mice, but its effect on the respiratory microbiota is unknown. We therefore aimed to observe the effects of QF on airway inflammation and respiratory microbiota in ovalbumin (OVA)-induced asthmatic mice. We also explored the potential mechanism of QF in reducing airway inflammation by regulating respiratory microbiota. Hematoxylin and eosin as well as periodic acid-Schiff staining were performed to observe the effects of QF on lung pathology in asthmatic mice. Cytokine levels in bronchoalveolar lavage fluid (BALF) specimens were also measured. Changes in respiratory microbiota were analyzed using 16S rRNA gene sequencing, followed by taxonomical analysis. In order to verify the metagenomic function prediction results, the expression of key proteins related to the MAPK and NOD-like receptor signaling pathways in the lung tissues were detected by immunohistochemistry. The current study found that QF had a significant anti-inflammatory effect in the airways of asthmatic mice. This is mainly attributed to a reduction in lung pathology changes and regulating cytokine levels in BALF. Analysis of the respiratory microbiota in asthmatic mice showed that the abundance of Proteobacteria at the phylum level and *Pseudomonas* at the genus level increased significantly and QF could significantly regulate the dysbiosis of respiratory microbiota in asthmatic mice. Metagenomic functional prediction showed that QF can downregulate the MAPK and Nod-like receptor signaling pathways. Immunohistochemical results showed that QF could downregulate the expression of p-JNK, p-P38, NLRP3, Caspase-1, and IL-1β, which are all key proteins in the signaling pathway of lung tissue. Our study therefore concluded that QF may reduce airway inflammation in asthmatic mice by regulating respiratory microbiota, and to the possibly downregulate MAPK and Nod-like receptor signaling pathways as its underlying mechanism.

## 1 Introduction

Bronchial asthma is a heterogeneous disease characterized by chronic airway inflammation and airway hyper-responsiveness. The major symptoms of asthma, ranging from mild symptoms to life-threatening attacks, include coughing, wheezing, shortness of breath, and chest tightness (Licari et al., 2018). Although asthma can occur at any age, it is particularly common in children. Epidemiological investigations have shown that the global incidence of asthma in children has increased from 11.1% to 13.2% over the last 10 years (Asher et al., 2020). At present, there are still a number of children worldwide with asthma that is not effectively controlled for various reasons (Murphy et al., 2020).

Airway inflammation is considered the most important pathophysiological process in asthma. T helper (Th) lymphocytes play an important role in airway inflammation (Tumes et al., 2017). Previous studies have shown that asthma can be divided into at least two distinct molecular phenotypes based on the degree of Th2 inflammation, which have been described as Th2-high and Th2-low (Robinson et al., 2017). Th2 cells produce interleukin (IL)-4, IL-13, and IL-5, eventually leading to the accumulation of eosinophils in the lungs (Finkelman et al., 2010). In recent years, studies have shown that severely asthmatic patients with a Th2-low phenotype have been noted to display predominantly neutrophilic inflammation of their airways (Ramakrishnan et al., 2019). Moreover, Th17 cells and their cytokines (IL-17 and IL-6, among others) are implicated in the development of neutrophilic inflammation. However, CD4^+^CD25^+^ regulatory T cells and their cytokines (IL-10 and TGF-β, among others) were significantly reduced in patients with asthma (Gandhi et al., 2020).

Following the development of high-throughput sequencing technology, the relationship between changes in the human microbiota and asthma has attracted increasing attention. The respiratory microbiota, which exhibits the mucosal surface of the respiratory tract, is a direct participant in the local mucosal immunity of the respiratory system and is closely related to the occurrence and development of asthma (Budden et al., 2019). Our recent study also found that dynamic changes in the respiratory microbiota are closely associated with the progression of chronic asthma, including airway inflammation and airway remodeling (Zheng et al., 2021). Huang et al. (2015) showed that an increase in Proteobacteria abundance in the respiratory tract can promote neutrophil aggregation, which may affect the patient’s response to corticosteroid therapy. A study of the composition of nasal microbiota showed that the increased abundance of *Streptococcus*, *Haemophilus*, and *Moraxella* was an important risk factor for predicting wheezing in preschoolers younger than 5 years, which persisted until a school-going age (Teo et al., 2018). In a mouse model study, *Prevotella* was found to significantly reduce neutrophil aggregation in the lung and production of Toll-like receptor (TLR) 2-mediated pro-inflammatory cytokines, thereby reducing lung inflammation, compared with *Haemophilus influenzae* (Larsen et al., 2015). In addition, some respiratory microbes can also play a role in reducing lung inflammation by producing metabolites (such as short-chain fatty acids) (Segal et al., 2017a; Segal et al., 2017b). These findings indicate that alterations in respiratory microbiota can affect the phenotype and severity of airway inflammation in patients with asthma by driving local mucosal immune responses. Furthermore, it is probable that the regulation of dysbiosis of respiratory microbiota can improve airway inflammation in asthma to some extent.

In recent years, an increasing number of studies have found that Traditional Chinese Medicine (TCM) compounds play a role in asthma prevention and treatment (Tsang et al., 2018; Cui et al., 2020). Qingfei oral liquid (QF), founded by Wang Shouchuan, a famous professor at Nanjing University of Chinese Medicine in China, has been used for the prevention and treatment of asthma in children for decades, and has achieved satisfactory results. It contains TCM concoction of *Ephedra sinica* Stapf [Ephedraceae], *Prunus armeniaca* var. Armeniaca [Rosaceae], and *Scutellaria baicalensis* Georgi [Lamiaceae], among others. A previous study by our team found that QF can reduce airway inflammation and airway hyper-responsiveness in respiratory syncytial virus (RSV)-infected asthmatic mice (Jing et al., 2020; Yu et al., 2021), but its effect on respiratory microbiota has not been observed. In view of this, the aim of the current study was to investigate the anti-inflammatory activities of QF and its effect on respiratory microbiota in mice with ovalbumin (OVA)-induced asthma and to explore the possible mechanism of QF in alleviating airway inflammation by regulating respiratory microbiota. The purpose of this study was to provide new knowledge for the prevention and treatment of asthma and provide a theoretical basis for the effective intervention of QF.

## 2 Materials and methods

### 2.1 Reagents and drugs

We obtained OVA from Sigma-Aldrich Chemical Co. (grade V; St. Louis, MO, United States). Mouse IL-4, IL-6, IL-10, and IL-17A ELISA kits and Imject Alum were purchased from Thermo Fisher Scientific (Rockford, IL, United States). Mouse total IgE and OVA-specific IgE ELISA kits were purchased from BioLegend (San Diego, CA, United States). Antibodies against NLRP3, Caspase-1, and IL-1β were obtained from Abcam Co. (Cambridge, MA, United States). Antibodies against p-P38, and p-JNK were obtained from CST Co. (Boston, MA, United States).

We obtained QF from the Chinese Pharmacy of Longhua Hospital, Shanghai University of Traditional Chinese Medicine. The standard dose of QF was 91 g (weight of total herb mixtures) and the composition of it was presented in Table 1. The extraction and quality control of QF were performed by the Department of Pharmacology at the Shanghai University of Traditional Chinese Medicine and Shanghai Fudan Fuda Science & Technology Co., Ltd. (Shanghai, China).

**TABLE 1:** Composition of Qingfei oral liquid (QF).

Latin name (Chinese name)	Parts & form used	Weight use (g)
Ephedra sinica Stapf (Ephedraceae) (Ma Huang)	Stem	3
Prunus armeniaca var. Armeniaca (Rosaceae) (Ku Xin Ren)	Dried ripe seed	10
Gypsum Fibrosum (Sheng Shi Gao)	Mineral	20
Scutellaria baicalensis Georgi (Lamiaceae) (Huang Qin)	Dried root	6
Morus alba L. (Moraceae) (Sang Bai Pi)	Dried bark of root	10
Lepidium apetalum Willd. (Brassicaceae) (Ting Li Zi)	Dried ripe seed	10
Kitagawia praeruptora (Dunn) Pimenov (Apiaceae) (Qian Hu)	Dried root	10
Reynoutria japonica Houtt. (Polygonaceae) (Hu Zhang)	Dried root and rhizome	12
Salvia miltiorrhiza Bunge (Lamiaceae) (Dan Shen)	Dried root and rhizome	10

### 2.2 UHPLC-MS/MS conditions

Samples were analyzed by suspending 100 mg of each compound in 10 ml of 50% methanol aqueous solution in a 15 ml centrifugal tube. The tube was then sonicated for 30 min, whereafter 1 ml of supernatant was centrifuged at 14,000 rpm for 5 min. Following centrifugation, the supernatant was filtered through a 0.22 µm microporous membrane, and placed into sample vials for UHPLC-MS/MS analysis. Control samples were processed under the same conditions.

The LC conditions were as follows. Column: ACQUITY UPLC HSS T3 column (2.1 × 100 mm, 1.8 μm); Column temperature: 35°C; Injection volume: 10 μl; Flow rate: 0.3 ml/min; Mobile Phase A: (deionized water with 0.1% formic acid); Phase B (acetonitrile with 0.1% formic acid). Specific gradient elution conditions were as follows: 0 min, 0% B; 10 min, 30% B; 25 min, 40% B; 30 min, 50% B; 40 min, 70% B; 45 min, 100% B; 60 min, 100% B; 60.5 min, 0% B and 70 min, 0% B.

The MS conditions were as follows. Q Exactive Orbitrap high resolution mass spectrometry was used to analyze sample. The detection mode was full MS-DDMS2, and positive and negative ion modes were scanned. Acquisition range: 100–1,200 Da, MS1 resolution: 70,000, MS2 resolution: 17,500, capillary voltage: ± 3.2 kV, capillary temp: 320°C, Aux gas heater temp: 350°C, sheath gas flow rate: 40 L/min, auxiliary gas flow rate: 15 L/min, AGC target: 1e6, TopN: 5, Full MS-ddMS2 NCE: 30, 40, 50.

### 2.3 Animal experiments

Female BALB/c mice (*n* = 36) aged 4–6 weeks and weighing 17.5–20.5 g were obtained from Shanghai Sippr-BK Laboratory Animal Co., Ltd. (Shanghai, China). All mice were maintained in a specific-pathogen-free grade animal room under controlled conditions with a 12 h light/dark cycle at a temperature of 24 ± 2°C with a relative appropriate humidity. Animal experiments were conducted in accordance with institutional guidelines for animal research. The Center for Laboratory Animals, Shanghai Children’s Hospital, School of Medicine, Shanghai Jiao Tong University, Shanghai, China authorized this experimental protocol (approval no. 2017Y003).

A mouse model of OVA-induced asthma was constructed as previously described, with slight modifications (Temelkovski et al., 1998). First, 36 mice were divided into two groups: control (*n* = 6) and OVA (*n* = 30). Mice in the OVA group were sensitized by intraperitoneal injection of 200 µl of a solution composed of 20 µg OVA dissolved in 100 µl sterile saline and 100 µl Imject Alum (containing 4 mg aluminum hydroxide (net weight)) on days 0, 7, and 14. Then, 30 mice from the OVA group were randomly divided into five groups: OVA group (no treatment, *n* = 6), OVA + QFH group (high-dose QF, *n* = 6), OVA + QFM group (medium-dose QF, *n* = 6), OVA + QFL group (low-dose QF, *n* = 6), and OVA + BUD group (budesonide aerosol inhalation, *n* = 6). For the OVA challenge, the mice were exposed to OVA aerosol (2.5% w/v) OVA solution in sterile saline administered using a PARI PRONEB Ultra compressor (Pari Proneb, Midlothian, WA, United States) for 30 min on days 21–24 and 28–31. The mice in the OVA + QFH group, the OVA + QFM group and the OVA + QFL group were given 1.82 g/d, 0.91 g/d and 0.455 g/d QF (Herbs dry weight), respectively by intragastric administration 30 min before OVA challenge. One hour prior to the OVA challenge, mice in the OVA + BUD group were nebulized with budesonide (1 mg budesonide in 3 ml normal saline) for 30 min. Mice in the OVA group (no treatment) were administered an equivalent volume of distilled water intragastrical, followed by nebulization with normal saline for 30 min before OVA challenge. Mice in the control group were injected intraperitoneally with saline and an Imject Alum emulsion and then exposed to an aerosol of sterile saline without OVA, according to the same schedule. The mice were sacrificed within 24 h of final nebulization. After the mice were euthanized, nasal lavage fluid (NLF), bronchoalveolar lavage fluid (BALF), and left lower lung tissue were collected and preserved.

### 2.4 Histological analysis and immunohistochemical staining of lung tissue

First, we ligated the left lower lung and removed it after complete bronchoalveolar lavage. The lungs were harvested and infused with 4% paraformaldehyde for 24 h. Sections (4 μm-thick) were embedded in paraffin and then subjected to hematoxylin and eosin (H&E) and periodic acid-Schiff (PAS) staining to evaluate airway inflammation and mucus production in the lung tissues. Pathological changes were observed under a light microscope at ×200 magnification. To grade the extent of lung inflammation and goblet cell hyperplasia, a semiquantitative scoring system was used as previously described (Kujur et al., 2015; Kubo et al., 2019).

To observe the expression levels of related proteins in lung tissues, ligated left lung tissues were fixed with 4% paraformaldehyde and embedded in paraffin. Sections (4 μm-thick) were deparaffinized and rehydrated. The sections were pretreated with 0.3% H_2_O_2_ in methanol for 15 min to quench the endogenous peroxide activity and boiled at 100°C for 20 min in a 10% citrate buffer to unmask the antigens. Sections were incubated in primary antibodies (anti-NLRP3 1:200, anti-Caspase-1 1:1,000, and anti-IL-1β 1:500, anti-p-P38 1:200, and anti-p-JNK 1:100) at 4°C overnight and stained with HRP-labelled anti-rabbit IgG (1:1,000). After washing, DAB substrate was applied to the sections. Representative images of each slide were acquired at ×200 magnification for morphometric and comparative analysis.

### 2.5 Collection of bronchoalveolar lavage fluid and nasal lavage fluid

After euthanasia of the mice by cervical dislocation, the lungs were washed four times with sterile saline, using endotracheal intubation to the lower respiratory tract (LRT) (0.5 ml per round) to obtain samples for BALF. Then, a sterile leather hose was reinserted to rinse the nasal cavity with normal saline (0.5 ml per round) for 3–4 times and to collect the NLF. Both BALF and NLF were filtered once with a 0.22 µm filter. The filtered BALF was centrifuged at 1,000 g for 15 min, and the supernatants were collected for cytokine detection. A filter membrane was used for DNA extraction. All specimens were stored at −80°C.

### 2.6 Cytokine and IgE measurement

All BALF specimens were collected as described in Section 2.5. The levels of total IgE and OVA-specific IgE, IL-4, IL-6, IL-17A, and IL-10 in BALF were measured using ELISA kits according to the manufacturer’s instructions.

### 2.7 16S rRNA gene sequencing and bioinformatics analysis

To explore the effect of QF on the respiratory microbiota of asthmatic mice, 16S rRNA gene sequencing was performed using NLF and BALF specimens from the control, OVA, OVA + QFM, and OVA + BUD groups. Total genomic DNA was extracted using the OMEGA Soil DNA Kit (Omega Bio-Tek, Norcross, GA, United States), according to the manufacturer’s instructions, and stored at −80°C until needed for analysis. The forward primer 338F (5-ACT​CCT​ACG​GGA​GGC​AGC​A-3) and reverse primer 806R (5-GGACTACHVGGGTWTCTAAT-3) were used to amplify the V3–V4 region of the bacterial 16S rRNA genes. After the individual quantification step, the amplicons were pooled in equal quantities and paired-end sequencing (2 × 300 bp) was performed using Illumina MiSeq (Illumina, San Diego, CA, United States) at Shanghai Personal Biotechnology Co., Ltd. (Shanghai, China). The procedures for DNA extraction, PCR amplification, and sequencing were based on previous studies^[10]^ of our team.

Microbiome bioinformatic analysis was performed using QIIME 2 2020.11 with minor modifications (Hall and Beiko, 2018). Briefly, raw sequence data were demultiplexed using the demux plugin, followed by primer cutting using the Cutadapt plugin. The sequences were subjected to quality filtering, denoising, and merging, and chimeras were removed using the DADA2 plugin (Callahan et al., 2016). Non-singleton amplicon sequence variants were used to construct a phylogeny using FastTree (Price et al., 2009). We used the q2-diversity plugin to compute different α diversity metrics using Pielou’s evenness indices and β diversity metrics using weighted UniFrac distance matrices. Principal coordinates analysis (PCoA) with weighted UniFrac distance matrices was used to study community composition. Taxonomy was assigned using a naive Bayes classifier pre-trained on the Greengenes 13_8_99% OTUs 16S rRNA gene full-length sequences and the q2-feature-classifier plugin (Bokulich et al., 2018). The bar chart of the composition of respiratory microbiota was completed using Wekemo Bioincloud (https://www.bioincloud.tech). Linear discriminant analysis effect size (LEfSe) (Segata et al., 2011) was used to detect differentially abundant taxa across groups using default parameters. The PICRUSt software package (Langille et al., 2013) was used for metagenomic functional prediction analysis. Kyoto Encyclopedia of Genes and Genomes (KEGG) pathways (Kanehisa et al., 2004) were used to identify metagenomic contents. LEfSe and PICRUSt analyses were performed using resources available at http://huttenhower.sph.harvard.edu/galaxy/. The STAMP software (Parks et al., 2014) was used to analyze the predicted metagenome and identify pathways associated with airway inflammation.

### 2.8 Statistical analysis

The expression levels of cytokines and related proteins were compared by analysis of variance using GraphPad Prism 8.0, and SPSS 24.0. The differences between the four groups were tested using one-way analysis of variance (ANOVA), and the variance between the two groups was compared using an Tukey’s multiple comparisons test. The Kruskal–Wallis test was used to estimate intergroup differences in α diversity metrics, β diversity metrics, and LEfSe analysis. The Wilcoxon test was used to compare subclasses. The predicted metagenome was analyzed using White’s non-parametric t-test for comparisons between the two groups. Statistical tests used in the study were two-sided, and a *p* value ≤0.05 was considered to indicate statistical significance.

## 3 Results

### 3.1 Composition of Qingfei oral liquid

**FIGURE 1:**
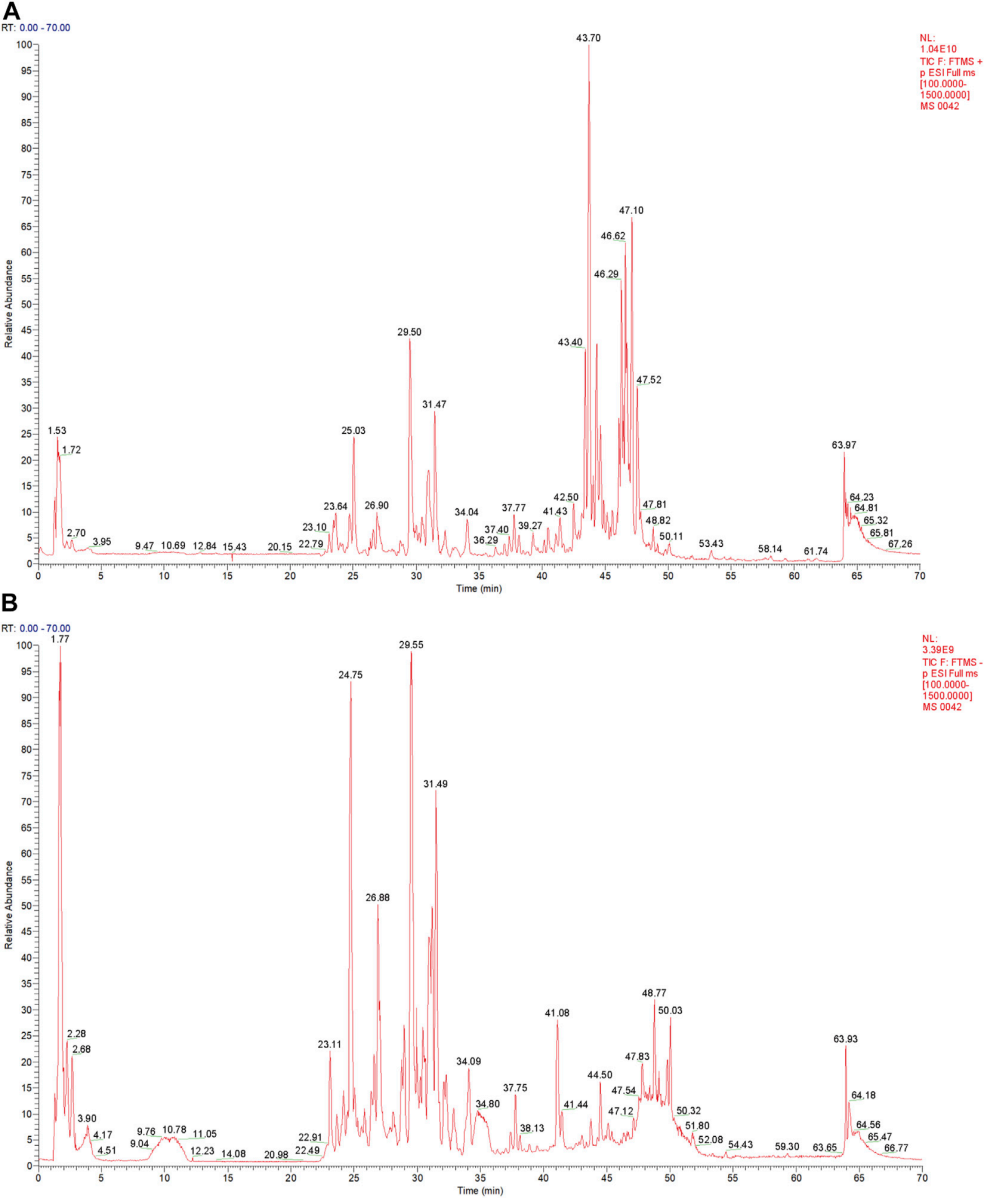
Total ion chromatography (TIC) of the extract of Qingfei oral liquid (QF). **(A)** TIC of the extract of QF in positive ion mode; **(B)** Total ion chromatography of the extract of QF in negative ion mode.

The QF extract was analyzed using UHPLC-MS/MS. The TIC in the positive and negative ion modes of the QF extract are shown in Figures 1A,B, respectively. Analysis of the top 20 compounds sorted by MS response values. The source of the active QF component was based on the Chinese Pharmacopoeia 2015 edition, the literature search was based on PubMed, and the compound data search was based on PubChem, Chemical Book, and SciFinder databases. The information on the top 20 compounds is presented in Table 2. The TIC of the blank samples are shown in Supplementary Figure S1.

**TABLE 2:** The information of top 20 compounds of the extract of Qingfei oral liquid (QF).

No.	Compound name	Formula	Mass deviation/ppm	Molecular weight	Rt/min	Match score	Area/×10^10^	Relative amount/%
1	Baicalin	C21H18O11	0.6	446.09	29.7	89.1	8.3	19.23
2	Wogonoside	C22H20O11	0.8	460.1	31.7	84.2	4.94	11.44
3	Amygdalin	C20H27NO11	0.6	457.16	24.9	93.6	4.36	10.11
4	Oroxylin A-7-O-β-D-glucuronide	C22H20O11	0.8	460.1	31.1	84.8	2.41	5.59
5	Cryptotanshinone	C19H20O3	1.3	296.14	44.8	87.4	1.91	4.42
6	Emodin-8-O-β-d-glucopyranoside	C21H20O10	0.2	432.11	31.4	92.9	1.57	3.63
7	Tanshinone IIA	C19H18O3	1.0	294.13	46.9	88.9	1.33	3.08
8	Sucrose	C12H22O11	0.2	342.12	1.9	93.6	1.29	3
9	Polydatin	C20H22O8	0.5	390.13	27.1	89.3	1.24	2.87
10	Emodin	C15H10O5	0.3	270.05	41.3	88.3	1.21	2.81
11	Praeruptorin C	C24H28O7	0.2	445.21	47.3	86.5	1.17	2.72
12	Praeruptorin A	C21H22O7	0.2	170.08	43.9	83.2	1.01	2.34
13	Baicalein	C15H10O5	0.4	270.05	34.2	88.6	0.89	2.06
14	Stachyose	C24H42O21	0.6	666.22	1.9	91	0.75	1.73
15	Chrysosplenetin B	C19H18O8	0.6	374.1	38.0	72.2	0.69	1.61
16	Decursinol	C14H14O4	0.5	246.09	41.6	76.3	0.63	1.46
17	Tinnevellin glucoside	C20H24O9	0.4	408.14	31.2	75.5	0.61	1.42
18	Resveratrol	C14H12O3	0.2	196.05	27.1	91.9	0.6	1.38
19	Wogonin	C16H12O5	0.3	284.07	37.6	92.6	0.44	1.03
20	Taxifolin	C15H12O7	0.3	304.06	26.5	86.1	0.42	0.96

### 3.2 Administration of Qingfei oral liquid attenuated airway inflammation in lung pathology in ovalbumin-induced asthmatic mice

**FIGURE 2:**
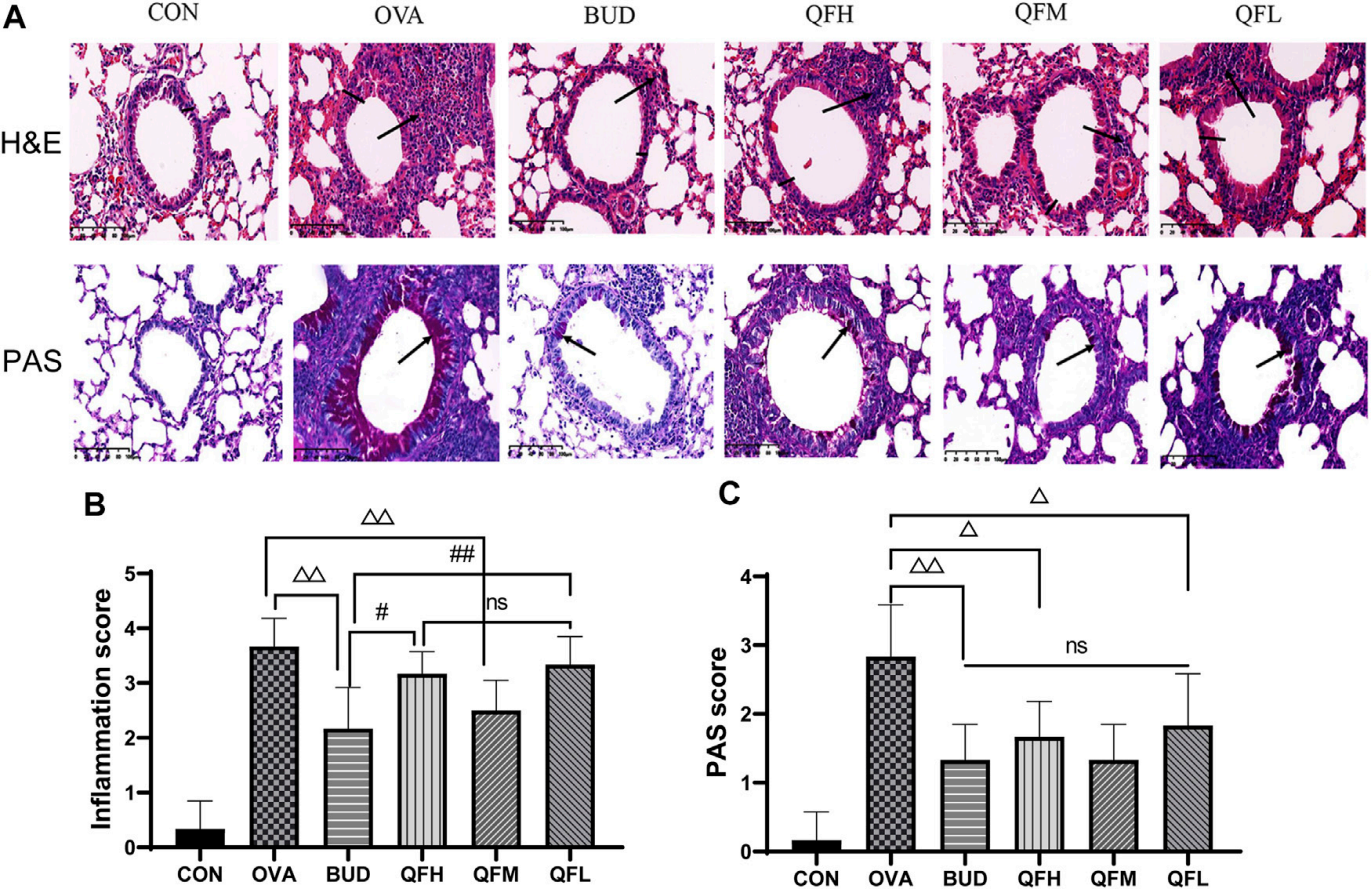
Effect of Qingfei oral liquid (QF) on pulmonary pathology in ovalbumin (OVA)-induced asthmatic mice. **(A)** Representative hematoxylin and eosin (H&E) staining were performed to observe airway inflammation in asthmatic mice (200×; the black arrows indicate the aggregation of inflammatory cells, and the black vertical line indicates the airway wall thickness). Periodic acid-Schiff (PAS) staining indicating the mucus-producing goblet cells around the small airways (200×; similar to the purple area indicated by the black arrow). **(B)** Inflammation scores are based on H&E staining. **(C)** The PAS scores indicating the mucus-producing goblet cells around the small airways. Data are expressed as mean ± standard deviation (SD) (one-way ANOVA followed by Tukey’s test). *n* = 6, ^△^ *p* ≤ 0.05 vs. the OVA group, ^△△^ *p* ≤ 0.01 vs. the OVA group; ^#^ *p* ≤ 0.05 vs. the budesonide (BUD) group, ^##^ *p* ≤ 0.01 vs. the BUD group, ns: no difference.

To investigate the effect of QF on airway inflammation in OVA-induced asthmatic mice, lungs were stained with H&E and PAS. Compared to control mice, OVA-challenged mice showed obvious inflammatory cell infiltration around the small airways, bronchial wall thickening, and constriction (Figures 2A,B). Treatment of OVA-challenged mice with QF and budesonide significantly alleviated airway inflammation. In the budesonide and QFM group, the number of inflammatory cells infiltrating the airway was significantly lower than in the OVA group, as was the airway wall thickness and the inflammation score. For OVA-challenged mice treated with QF, medium-dose QF had the best effect (Figures 2A,B). PAS staining showed that the number of mucus-secreting goblet cells increased in OVA-challenged mice compared to that in control mice (Figures 2A,C). The budesonide and QF groups significantly reduced airway mucus secretion when compared to the OVA group at different doses, but there was no significant difference in the PAS score between the treatment groups. We can deduce that QF and budesonide alleviated these pathological changes. These results showed that QF can significantly alleviate airway inflammation in the lung pathology of asthmatic mice.

### 3.3 Administration of Qingfei oral liquid decreased inflammatory cytokines in bronchoalveolar lavage fluid of mice with ovalbumin-induced asthma

**FIGURE 3:**
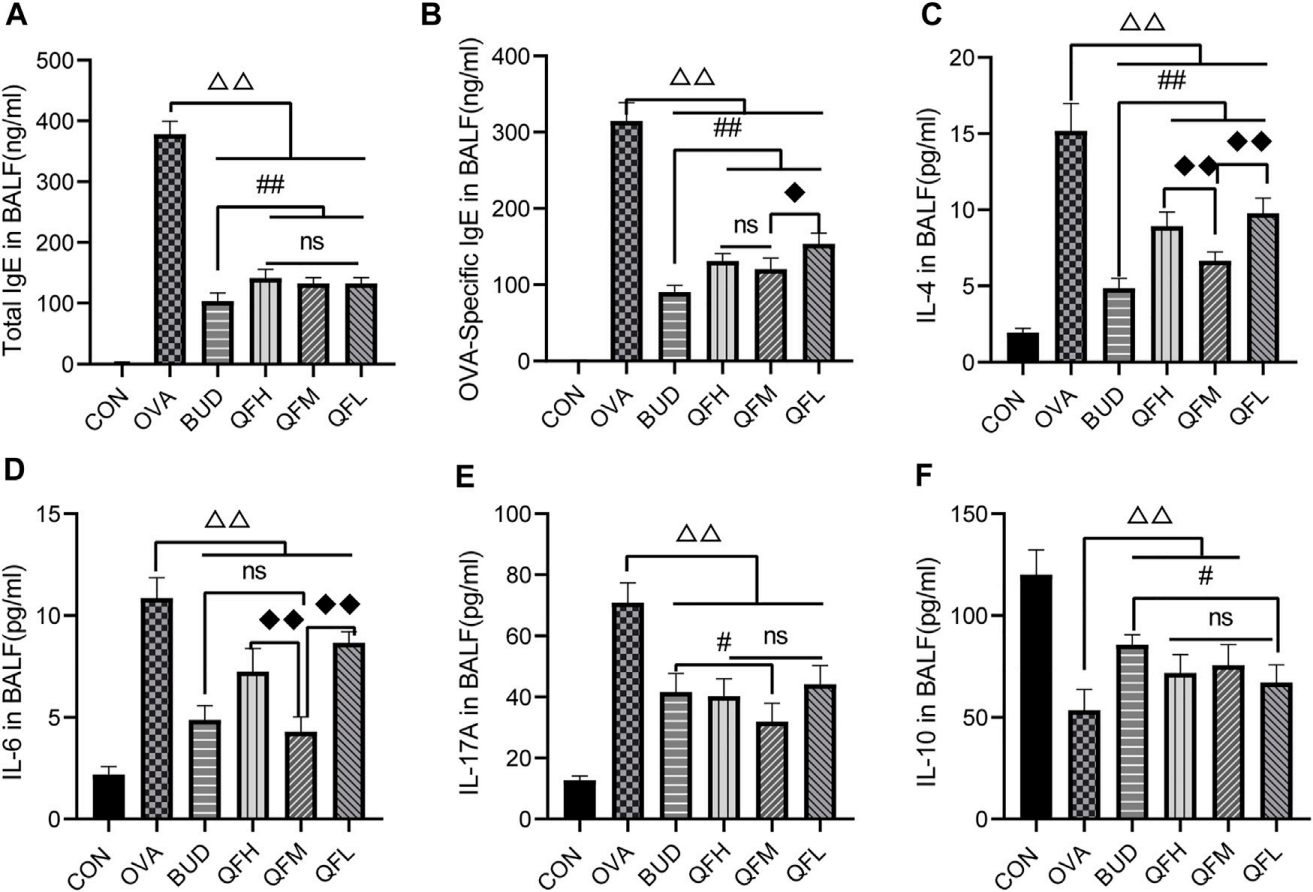
Effects of Qingfei oral liquid (QF) on IgE and inflammatory cytokines in bronchoalveolar lavage fluid (BALF) of asthmatic mice. BALF samples from different groups were collected to detect the levels of **(A)** total IgE, **(B)** Ovalbumin (OVA)-specific IgE, **(C)** IL-4, **(D)** IL-6, **(E)** IL-17A, and **(F)** IL-10 using ELISA. Data are expressed as mean ± standard deviation (SD) and were performed with one-way ANOVA followed by Tukey’s tests. *n* = 6, ^△^ *p* ≤ 0.05 vs. the OVA group, ^△△^ *p* ≤ 0.01 vs. the OVA group; ^#^ *p* ≤ 0.05 vs. the budesonide (BUD) group, ^##^ *p* ≤ 0.01 vs. the BUD group; ^◆^ *p* ≤ 0.05 vs. the QFM group, ^◆◆^ *p* ≤ 0.01 vs. the QFM group; ns: no difference; line over bars indicates that all groups are include.

Total IgE, OVA-specific IgE, IL-4, IL-6, IL-17A, and IL-10 levels in BALF were measured using ELISA (Figure 3). Compared with the control group, the OVA group had significantly higher levels of total IgE (Figure 3A), OVA-specific IgE (Figure 3B), IL-4 (Figure 3C), IL-6 (Figure 3D), and IL-17A (Figure 3E), but lower levels of IL-10 (Figure 3F). Similar to the OVA-challenged mice treated with budesonide, the levels of total IgE, OVA-specific IgE, IL-4, IL-6, and IL-17A in the BALF of OVA-challenged mice treated with QF were significantly decreased (Figures 3A–E). Both QF and budesonide-treated mice showed increased levels of IL-10 to some extent (Figure 2F). In addition, QF was less effective than budesonide at reducing the levels of total IgE, OVA-specific IgE, and IL-4 (*p* < 0.05). QFM showed no significant difference in reducing IL-6 levels or increasing IL-10 levels when compared to budesonide (*p* > 0.05), but significantly improved in reducing IL-17A levels (*p* < 0.05). The effect of QF on reducing IL-6, IL-4, and IL-17A levels was especially significant in mice treated with intermediate doses of QF. These results suggest that QF regulates cytokine levels in the BALF of OVA-induced asthmatic mice.

### 3.4 Qingfei oral liquid altered the diversity of the respiratory microbiota in mice with ovalbumin-induced asthma

**FIGURE 4:**
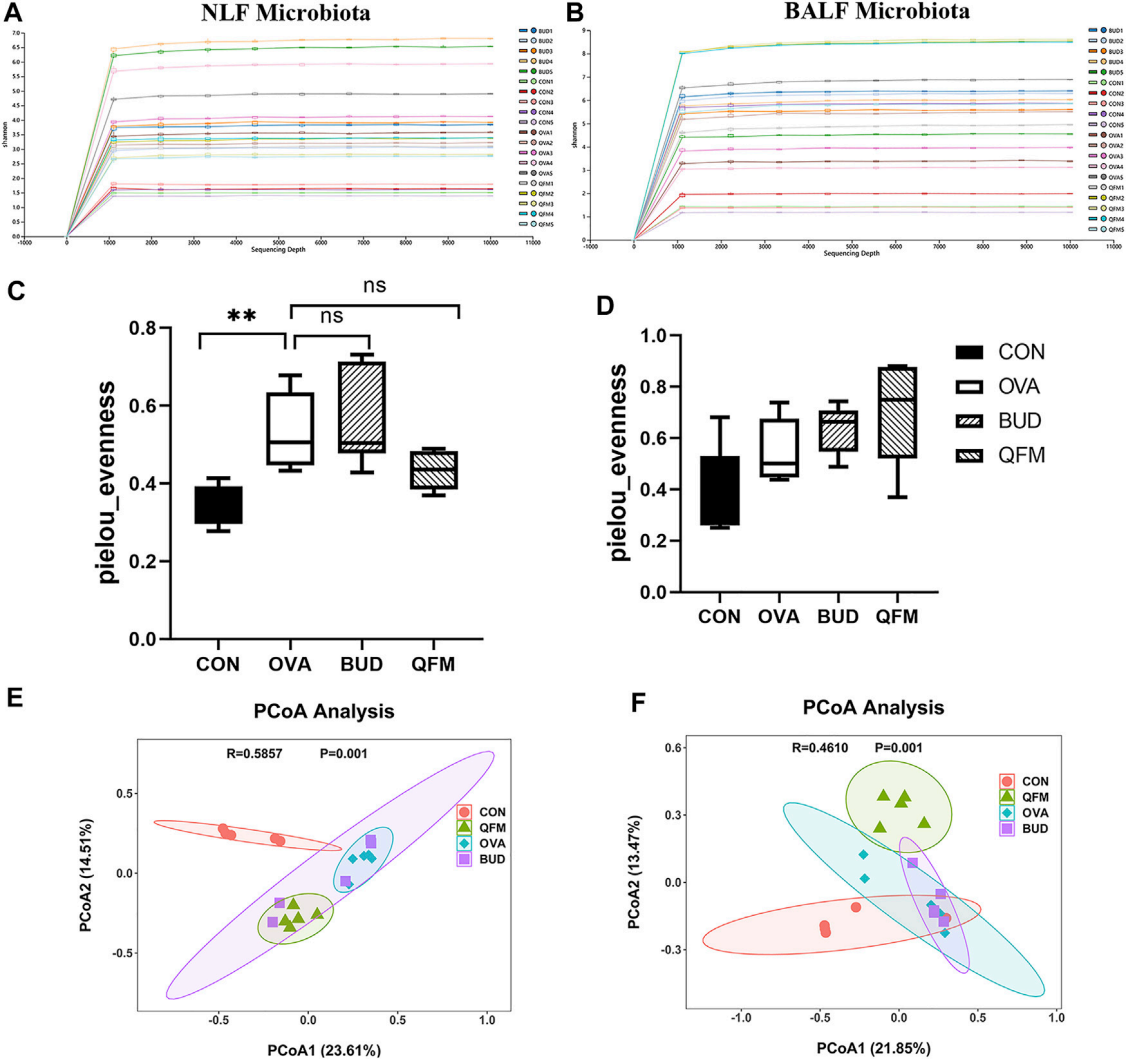
Effects of Qingfei oral liquid (QF) on the diversity of the respiratory microbiota in ovalbumin (OVA)-induced asthmatic mice. **(A)** The alpha rarefaction curve in nasal lavage fluid (NLF) microbiota. **(B)** The alpha rarefaction curve in bronchoalveolar lavage fluid (BALF) microbiota. **(C)** α diversity analysis (using the Pielou’s evenness) of the NLF microbiota. **(D)** α diversity analysis (using the Pielou’s evenness) of the BALF microbiota. **(E)** PCoA plot showing the β diversity of NLF microbiota (*p* = 0.001). **(F)** PCoA plot showing the β diversity of BALF microbiota (*p* = 0.001). PCoA of all samples using weighted UniFrac distance. PCoA, principal coordinates analysis. (*n* = 5 in each group).

Since the mid-dose QF had the best anti-airway inflammation effect, this study further compared the differences in respiratory microbiota among the control, OVA, OVA + QFM (QFM), and OVA + BUD (BUD) groups. The shape of the alpha rarefaction curve indicated that the sequencing depth was sufficient (Figures 4A,B). The α diversity represents the diversity of microbial groups in the model, and the β diversity analysis indicated the microbial diversity in different groups of mice. The upper respiratory tract (URT) microbiota (NLF samples) of the OVA-induced mice had a significantly higher α diversity (*p* < 0.05) than that of the control mice, as determined using Pielou’s evenness (Figure 4C). The URT microbiota of OVA-challenged mice treated with QF or budesonide did not differ significantly from that of the untreated OVA-challenged mice. However, there was no significant difference in the Pielou’s evenness indices of the LRT microbiota (BALF samples) among the control, OVA, QFM, and BUD groups (*p* > 0.05) (Figure 4D).

The weighted UniFrac distance was used to evaluate β-diversity among the different groups. The scatter plot based on the PCoA scores indicated a clear separation of community composition between the control and OVA groups. PCoA showed that the β-diversity of the respiratory microbiota of OVA-induced mice treated with QF was significantly different from that of untreated OVA-challenged mice (Figures 4E,F). These results indicate that QF can affect the diversity of the respiratory microbiota in OVA-induced asthmatic mice.

### 3.5 Qingfei oral liquid regulated the composition of the respiratory microbiota in mice with ovalbumin-induced asthma

**FIGURE 5:**
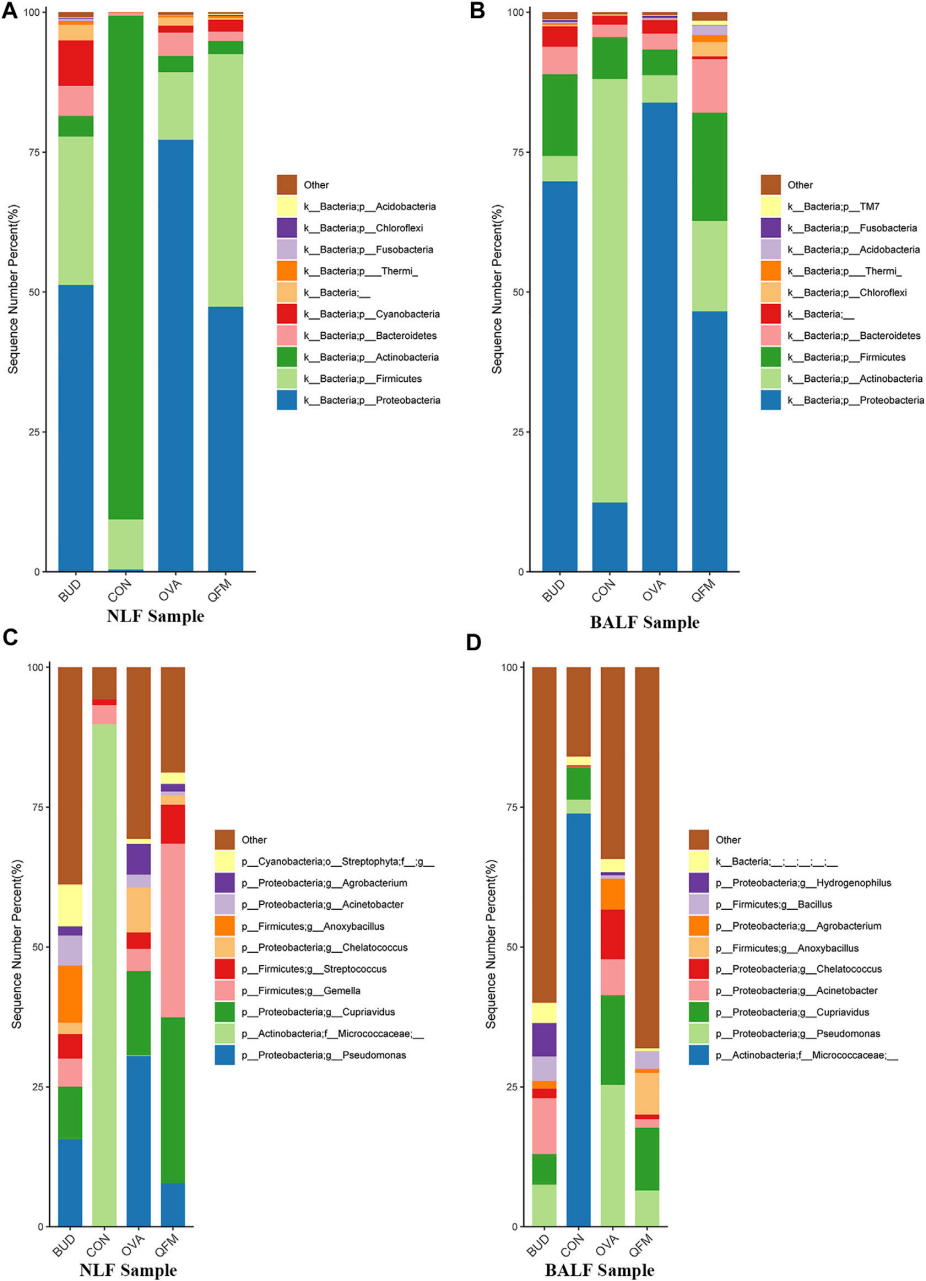
Effects of Qingfei oral liquid (QF) on the composition of respiratory microbiota in ovalbumin (OVA)-induced asthmatic mice. **(A)** The composition of nasal lavage fluid (NLF) microbiota at the phylum level. **(B)** The composition of bronchoalveolar lavage fluid (BALF) microbiota at the phylum level. **(C)** The composition of NLF microbiota at the genus level. **(D)** The composition of the BALF microbiota at the genus level. (*n* = 5 in each group; only the top 10 legends with high abundance are shown).

To understand the effect of QF on the composition of the respiratory microbiota in OVA-induced asthmatic mice, we analyzed the microbial composition at the phylum and genus levels. At the phylum level, Actinobacteria was predominant in the control group, whereas Proteobacteria was the most abundant phylum in the OVA group (in both URT and LRT samples). Fortunately, Proteobacteria were significantly reduced in OVA-induced asthmatic mice treated with QF and budesonide, and Actinobacteria were significantly increased in OVA-induced asthmatic mice treated with QF (Figures 5A,B).

At the genus level, Micrococcaceae was most abundant in the control group, whereas *Pseudomonas* and *Cupriavidus* were more abundant in the OVA group (Figures 5C,D). In asthmatic mice treated with QF and budesonide, the proportion of *Pseudomonas* decreased significantly. *Cupriavidus* decreased significantly in asthmatic mice treated with budesonide, but there was no significant difference between QF and untreated asthmatic mice.

**FIGURE 6:**
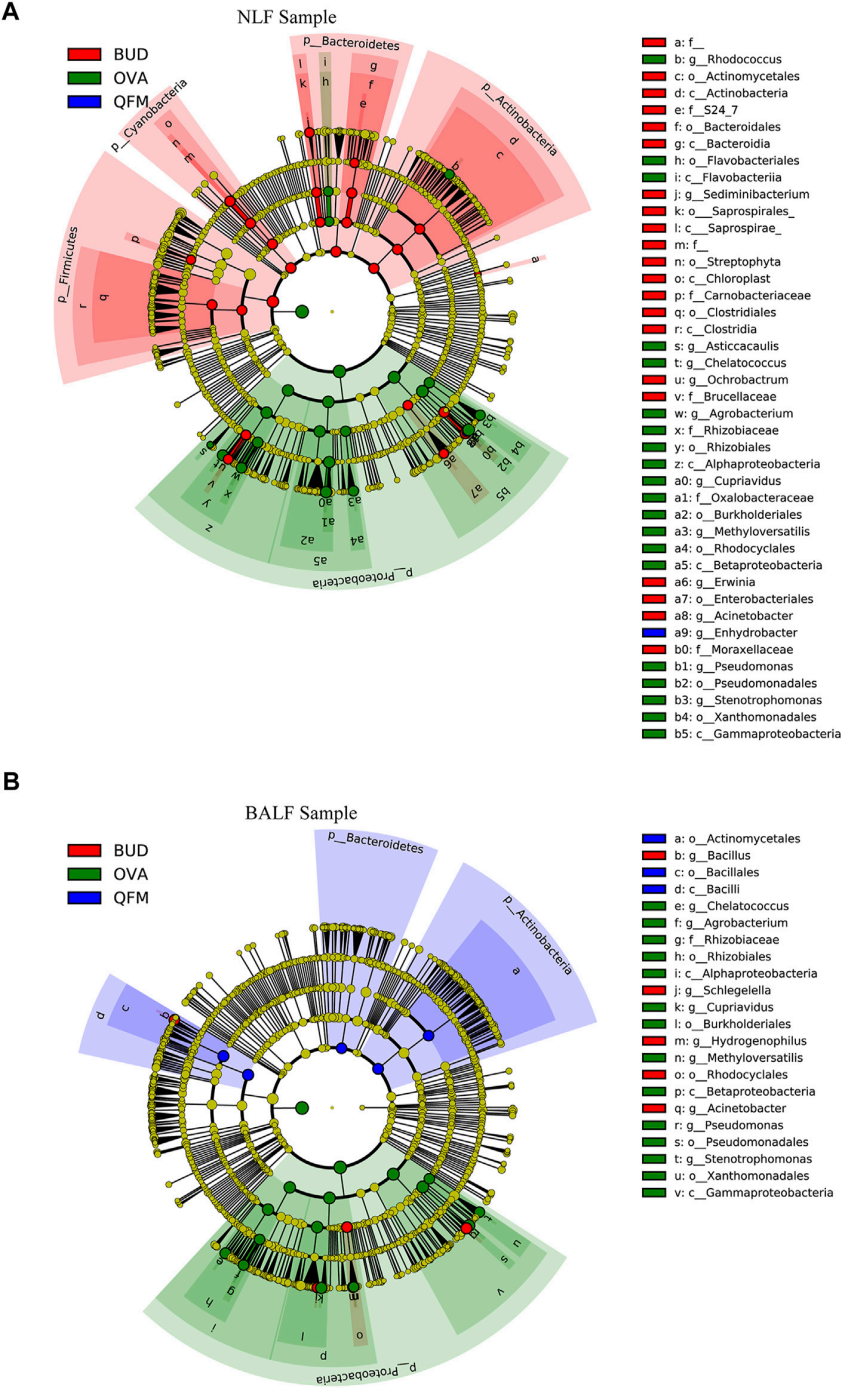
Different abundances of bacterial communities in the respiratory samples, as indicated in LEfSe analysis. The differences are indicated by the color of over-represented taxa: green indicating ovalbumin (OVA)-induced mice, blue indicating Qingfei oral liquid medium concentration (QFM) treated mice, and red indicating budesonide (BUD) treated mice. **(A)** Different abundances of bacterial communities in the upper respiratory tract (URT) (nasal lavage fluid (NLF) samples) with LDA scores >2.5. **(B)** Different abundances of bacterial communities in the lower respiratory tract (LRT) (bronchoalveolar lavage fluid (BALF) samples) with LDA scores >3.0. The circles represent phylogenetic levels from phylum (innermost circle) to genera (outermost circle). *n* = 5 in each group; adjusted *p* values ≤0.05.

To further identify the effect of QF on the composition of the microbiota in OVA-induced asthmatic mice, we analyzed the different abundances of bacterial communities using LEfSe (Figure 6). Analysis of microbial composition at the genus level using LEfSe revealed that among the URT and LRT microbiota, *Pseudomonas*, *Cupriavidus* and many other genera from Proteobacteria were present in the OVA group, *Bacilli* from Firmicutes in the QFM group, and *Bacillus* from Firmicutes in the BUD group. These results indicate that QF can regulate the composition of respiratory microbiota in asthmatic mice to some extent.

### 3.6 Effects of Qingfei oral liquid on metagenomic functional prediction of the respiratory microbiota of mice with ovalbumin-induced asthma

**FIGURE 7:**
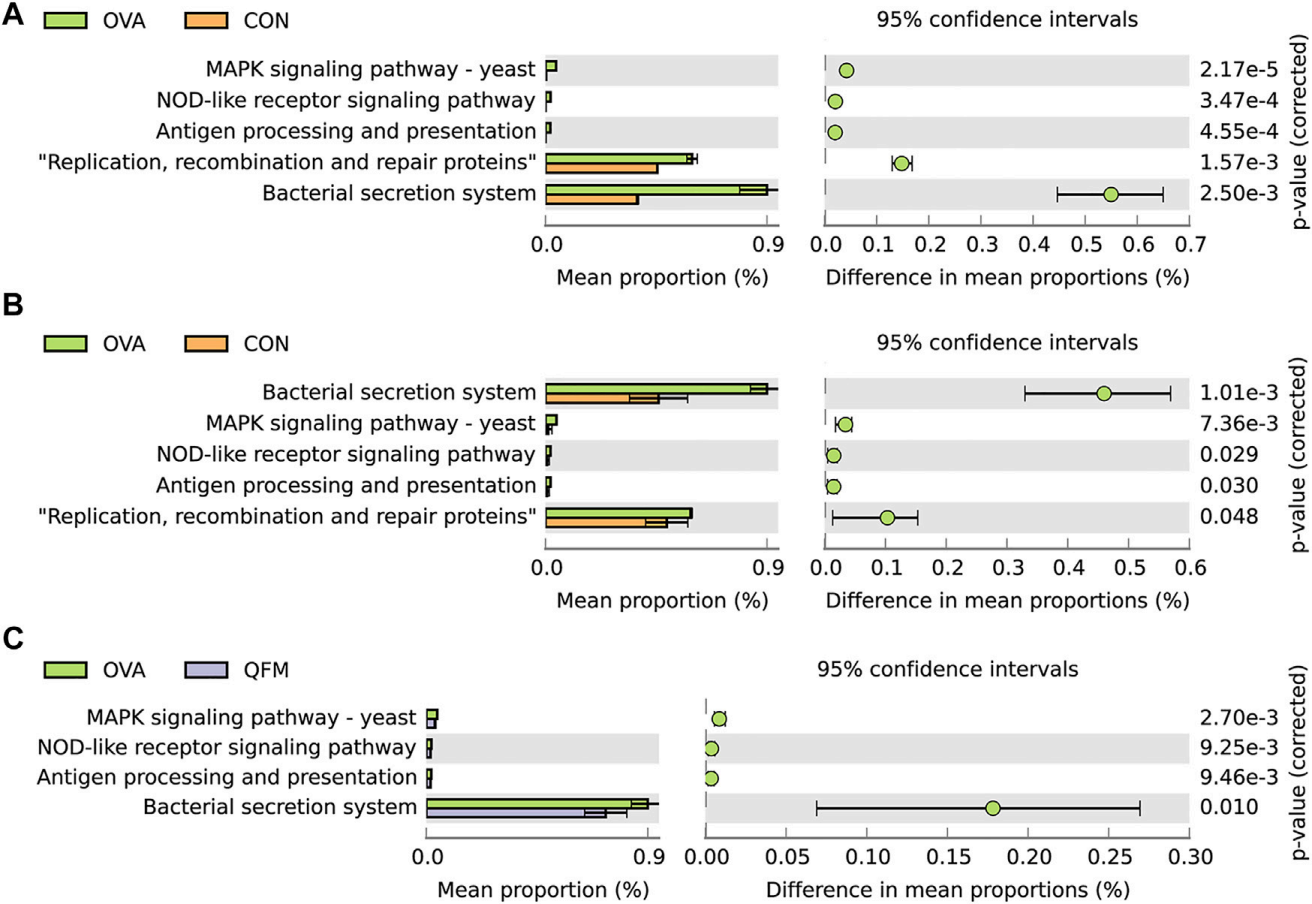
Effects of Qingfei oral liquid (QF) on metagenomic functional prediction of the respiratory microbiota in ovalbumin (OVA)-induced asthmatic mice. Bacterial metagenomic functional categories were derived from Level 3 KEGG pathways. **(A)** Control group vs. the OVA group for the nasal lavage fluid (NLF) microbiota. **(B)** Control group vs. the OVA group in bronchoalveolar lavage fluid (BALF) microbiota. **(C)** BALF microbiota of the OVA group vs. the QF medium (QFM) concentration group. Gene functions with significant differences (corrected *p*-value ≤ 0.05, White’s non-parametric t test in STAMP) and parts of the pathways associated with asthma are shown.

To understand whether alterations in the respiratory microbiota contribute to airway inflammation in asthmatic mice, we performed bacterial metagenomic function prediction analyses using the PICRUSt program. We explored the differences in the bacterial metagenomic functional prediction among the different groups at KEGG Level 3 (Figure 7).

In both URT and LRT samples, the MAPK signaling pathway-yeast, NOD-like receptor signaling pathway, antigen processing and presentation, bacterial secretion system, and replication, recombination, and repair proteins were significantly upregulated in the OVA group compared to the control group (Figures 7A,B). Among these pathways, MAPK signaling pathway-yeast and NOD-like receptor signaling pathway have been shown to be closely associated with airway inflammation. In LRT samples, MAPK signaling pathway-yeast and Nod-like receptor signaling pathway were significantly downregulated in QF treated asthmatic mice, but no similar changes were found in URT samples (Figure 7C). Therefore, we speculated that the underlying mechanism of QF to improve airway inflammation in OVA-induced asthmatic mice by regulating the respiratory microbiota may be related to the down-regulation of MAPK signaling pathway-yeast and NOD-like receptor signaling pathway.

### 3.7 Qingfei oral liquid regulated the expression of key proteins related to MAPK and NOD-like receptor signal pathway in ovalbumin-induced asthmatic mice

Metagenomic functional prediction showed that QF could downregulate the MAPK and Nod-like receptor signaling pathways related to airway inflammation. MAPK is an important transmitter of signals from the cell surface to the nucleus. Three distinct MAPK pathways have been described: p38 MAPK pathway, c-Jun amino-terminal kinase (JNK) pathway, and extracellular signal-regulated kinases (ERKs or p42/44 MAPK pathway). The JNK and p38 MAPK pathways, known as stress-activated protein kinases, respond to inflammatory and environmental physical insults, whereas the ERK pathway is activated by mitogenic and proliferative stimuli (Khorasanizadeh et al., 2017; Manley et al., 2019). The Nod-like receptor family pyrin domain-containing protein 3 (NLRP3) inflammasome is an important component of innate immunity that can recognize intracellular pathogens or injury-related molecular patterns and mediate the processing, maturation, and release of a variety of cytokines, including Il-1β, are involved in the regulation of the occurrence and development of various inflammatory diseases. The NLRP3 inflammasome is mainly composed of NLRP3, apoptosis-associated speck-like protein containing CARD (ASC) and caspase-1 (Gritsenko et al., 2020; Louvrier et al., 2020).

**FIGURE 8:**
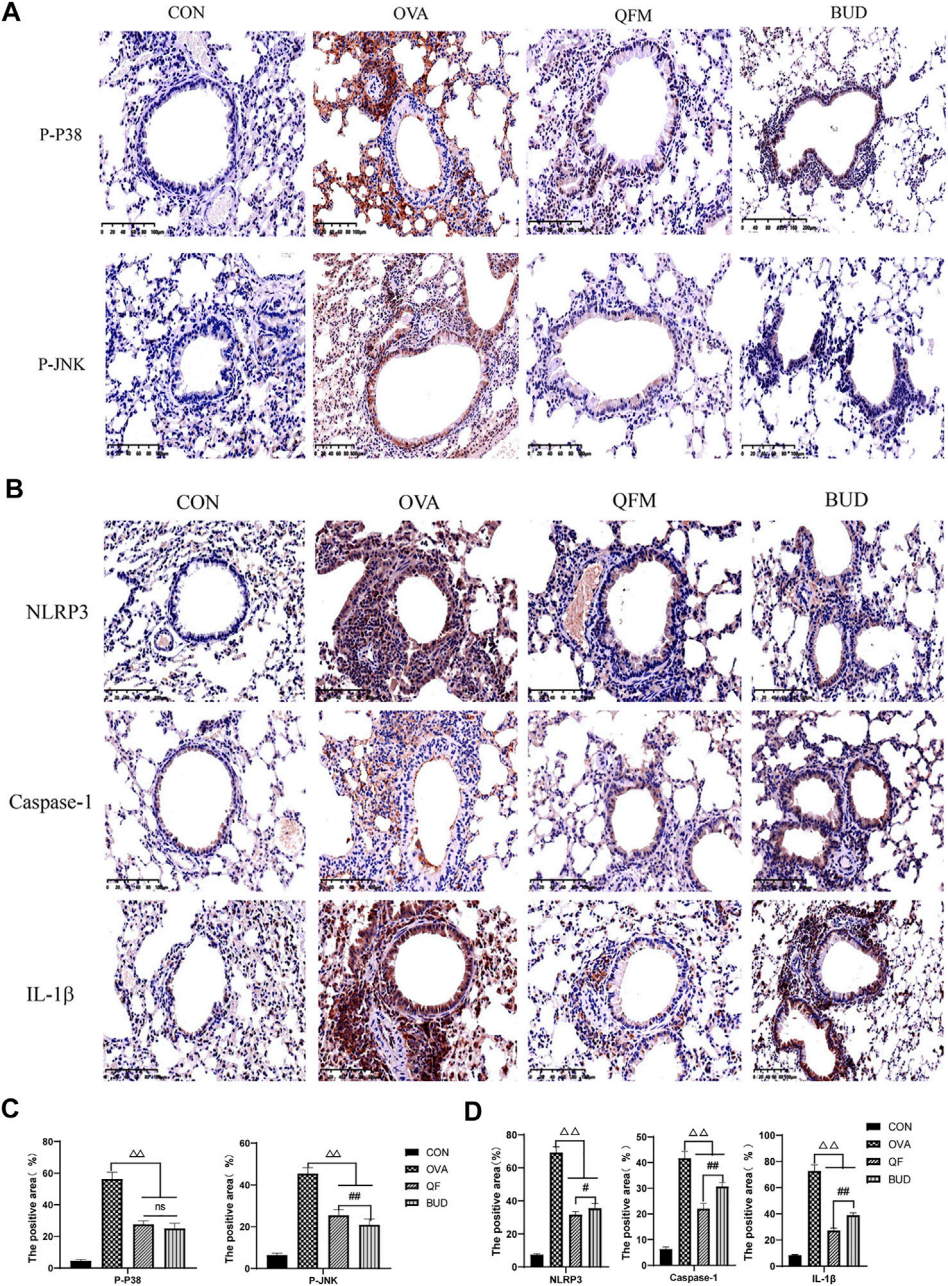
Effects of Qingfei oral liquid (QF) on the expression of key proteins related to MAPK and NOD-like receptor signal pathway. **(A)** Representative immunohistochemistry staining of p-P38 and p-JNK (200×, Brown particles represent positive protein expression). **(B)** Representative immunohistochemistry staining of NLRP3, caspase-1, and IL-1β (precursor IL-1 β and cleaved IL-1 β) (200×, Brown particles represent positive protein expression). **(C)** The positive area of p-P38 and p-JNK (%). **(D)** The positive area of NLRP3, caspase-1, and IL-1β (%). Each bar represents the mean ± standard deviation (SD). For all of these key proteins, there were significant differences between the control and the OVA groups (*p* ≤ 0.01). (*n* = 6, ^△^ *p* ≤ 0.05 vs. the OVA group, ^△△^ *p* ≤ 0.01 vs. the OVA group; ^#^ *p* ≤ 0.05 vs. the budesonide (BUD) group, ^##^ *p* ≤ 0.01 vs. the BUD group; ns: no difference; one-way ANOVA followed by Tukey’s test).

Therefore, the expression levels of p-P38, p-JNK and NLRP3, Caspase-1, IL-1β in the lung tissues were measured using Immunohistochemistry to confirm the prediction results (Figure 8). Compared with the control mice, the expression levels of p-P38 and p-JNK were significantly increased in the lung tissues of OVA-induced asthmatic mice but significantly decreased in the lung tissues of asthmatic mice treated with QF (Figure 8A). The effect of QF on reducing p-JNK expression levels was less efficient than BUD treatment (*p* < 0.01), whereas the effect on reducing p-P38 expression levels was not significantly different from BUD (*p* > 0.05; Figure 8C). Similarly, compared to the control mice, mice with OVA-induced asthma showed a significantly higher expression of NLRP3, Caspase-1, IL-1β in the lung tissues, and the expression of these key proteins was significantly decreased in asthmatic mice treated with QF (Figure 8B). When compared to BUD treatment, QF had more effect on lowering NLRP3, Caspase-1 and IL-1β expression levels (Figure 8D). These results further confirmed the metagenomic functional prediction of QF in the respiratory microbiota.

## 4 Discussion

In the present study, we explored the anti-inflammatory activities of QF and its influence on respiratory microbiota in mice with OVA-induced asthma. We found that the downregulation of MAPK and Nod-like receptor signaling pathways may be the underlying mechanism of QF which alleviates airway inflammation in asthma by regulating respiratory microbiota. Furthermore, we also identified and analyzed bioactive compounds in QF, including baicalin, wogonoside, amygdalin, cryptotanshinone, polydatin, emodin, resveratrol, and baicalein, among others, using UHPLC-MS/MS. Previous studies have shown that these compounds have anti-inflammatory (He et al., 2020), anti-oxidation (Nagappan et al., 2019), anti-allergic, spasmolytic, immune response inhibition (Bui et al., 2017; Son et al., 2021) and other effects.

Our study found that QF can significantly reduce the aggregation of inflammatory cells around the small airway, thickening of the bronchial wall, and secretion of mucus in the lung tissue of OVA-induced asthmatic mice, and also reduce the levels of IgE, IL-4, IL-6, and IL-17A in BALF. The best effect was delivered by QF at a medium dose. QF has been widely used in the treatment of pediatric respiratory diseases in China and has been approved by the Chinese National Drug administration (Lin et al., 2021). Children aged about 6 years old should take one dose (crude drug 91 g) daily, which is equivalent to the medium dose of mice according to the conversion method of body surface area between mice and human. In our study, the high-dose and low-dose groups were set up respectively, that is, the dose of the medium-dose group was doubled, and the dose of the medium-dose group was halved. The high-dose group was set to see if increasing the dose would lead to better results. The experimental results showed that the high dose group could not improve the clinical efficacy in OVA-induced asthmatic mouse models. From the composition of Qingfei oral liquid, there are some herbs that affect the metabolism of bacteria. High dose of QF might affect some beneficial bacteria in respiratory tract, so the effect is reduced. It is suggested that there is no need to increase the dose of QF in clinical application to improve the efficacy. Previous research by other members of the research team found that QF alleviated asthma exacerbation by decreasing the levels of IL-4, IL-6, and IL-13 in the serum and inflammatory cells in the lung tissue of RSV-infected asthmatic mice (Zhou et al., 2018; Yu et al., 2021). In this study, QF was comparable to budesonide in reducing IL-6 and IL-17A and increasing IL-10 levels, but not as effective as budesonide in reducing IgE and IL-4 levels. Numerous previous studies (Kuo et al., 2017; Lambrecht et al., 2019; Hinks et al., 2021) have shown that eosinophilic asthma is mainly characterized by IgE and Th2 cytokines (IL-4, IL-5, etc.), whereas neutrophil asthma is mainly characterized by Th17 cytokines (IL-17, IL-6, etc.), which are involved in the development of steroid-resistant asthma (Nabe, 2020). In this study, a mouse model of OVA-induced asthma was dominated by eosinophil inflammatory infiltration. This is based on the increased the total IgE, OVA-specific IgE, and IL-4 levels as shown in Figure 3, and the highest proportion of eosinophils in BALF in Supplementary Figure S2. Therefore, the mouse model of OVA-induced asthma is mainly Th2-driven inflammation in this study. Interestingly, genetic predisposition and microbial metabolites appear to influence T cell differentiation plasticity. Several recent studies have linked the pathogenesis of asthma caused by IgE and IL-4 to a TH17-dependent mechanism. LPS stimulation promotes the transition from Th2-derived airway eosinophil inflammation to Th17-derived neutrophil inflammation in an ovalbumin allergy mouse model of asthma (Zhao et al., 2017), and IgE-related polymorphisms affect asthma TH 17 gene expression (Worth et al., 2018). Furthermore, IL4R variants linked to allergic asthma were discovered to exacerbate airway inflammation by promoting regulatory T cell conversion to TH17-like cells (Massoud et al., 2016). Numerous studies have shown that allergic asthma can progress to IL-17-mediated neutrophilic asthma. Even so, as the inflammation persisted, neutrophil inflammation mediated by Th17 cells and cytokines began to play a role in the asthma process, due to the persistence of inflammation, neutrophil inflammation mediated by Th17 cell and cytokines also began to participate in the process of asthma. In our previous study (Zheng et al., 2021), dynamic observation of OVA-induced asthmatic mouse models showed that the 2-week phase of nebulization was the transition from eosinophilic inflammation to neutrophil inflammation and the initiation of small airway remodeling, so we selected the asthmatic mouse model during this critical transition period for our study. Many previous studies (Lönnkvist et al., 2001; Wenzel et al., 2016) have shown that Budesonide has a unique advantage for eosinophil asthma. In our asthmatic mouse model, it was found that Budesonide had a stronger inhibitory effect on eosinophilic inflammation than Qingfei oral Liquid (QF), but Traditional Chinese Medicine (TCM) compounds QF had a stronger inhibitory effect on IL-17 induced neutrophil inflammation than Budesonide. These results suggest that QF may have some advantages in the treatment of neutrophil asthma.

Our previous studies found that changes in respiratory microbiota are closely related to the pathophysiological processes of asthma, including airway inflammation (Zheng et al., 2021). Therefore, we proposed the following hypothesis: QF alleviates airway inflammation by regulating the respiratory microbiota of asthmatic mice. We found that QF and budesonide had no significant effect on the α-diversity of the respiratory microbiota in asthmatic mice, but both could affect the β-diversity of the respiratory microbiota to some extent. Since QF can alter the β-diversity of the respiratory microbiota composition in asthmatic mice, we further explored its effect on the composition of the respiratory microbiota. At the phylum level, compared to normal mice, the abundance of Proteobacteria in asthmatic mice was significantly increased, while that of Actinobacteria was significantly decreased. Previous studies have shown a significant increase in Proteobacteria in patients with asthma, especially in severe or steroid-resistant asthma (Li et al., 2017; Taylor et al., 2018). Actinobacteria were found to be more abundant in the normal population and in patients with eosinophilic asthma (Li et al., 2017; Hufnagl et al., 2020). Surprisingly, QF significantly reduced the abundance of Proteobacteria and slightly increased the abundance of Actinobacteria in the asthmatic mice. This may be one of the mechanisms through which QF reduces neutrophil inflammation. At the genus level, the abundance of *Pseudomonas* was significantly higher in asthmatic mice than in normal mice, while QF significantly reduced the abundance of *Pseudomonas*. Ferri et al. (2020) showed that the presence of *Pseudomonas* in sputum is an important risk factor for the persistent and frequent exacerbation of asthma. These results suggest that QF can regulate the composition of the respiratory microbiota in asthmatic mice.

Therefore, what effect does QF have on the host by regulating the dysbiosis of the respiratory microbiota in asthmatic mice? We explored the differences in bacterial metagenomic functional predictions. The results showed that the “MAPK signaling pathway-yeast” and “NOD-like receptor signaling pathway” related to airway inflammation were significantly upregulated in asthmatic mice, and QF significantly downregulated these two signaling pathways in the LRT. The three subfamilies of MAPKs have been involved in the pathogenesis of asthma, and JNK and P-38, in particular, are more closely associated with asthmatic airway inflammation, which can cause high expression of downstream factors such as IL-6, IL-1β, and IL-4 and IL-5, respectively (Dong et al., 2002; Pahl et al., 2002; Khorasanizadeh et al., 2017). The phosphorylation states and/or activities of all three MAPK members are upregulated in animal models of asthma (Dong et al., 2002). Inhibitors of JNK and P-38 both can significantly reduce airway inflammation in asthmatic patients (Bhavsar et al., 2010; Khorasanizadeh et al., 2017). NOD-like receptors (NLRs; nucleotide-binding oligomerization do-main-like receptors) represent a class of widespread, sophisticated signaling regulators, including more than 20 members have been reported (Liu et al., 2019). NLRP3 belongs to the NLRP subfamily, which is an inflammatory protein complex composed of the intracellular innate immune receptor NLRP3, adaptor protein ASC, and protease caspase-1 (Guo et al., 2015). They can help the body recognize endogenous and exogenous abnormal substances and release the inflammatory factors IL-1β and IL-18 (Tang et al., 2019). Previous studies have shown that elevated caspase-1 and IL-1β can be detected in mouse models of asthma, and increased IL-18 can also be detected in the sputum of asthmatic patients and is associated with asthma severity (Harada et al., 2009; Liao et al., 2015).

In order to verify the metagenomic function prediction results, this study further explored the expression of key proteins related to MAPK and NOD-like receptor signaling pathways in the lung tissues. The expression levels of p-P38 and p-JNK were significantly increased in the lung tissues of asthmatic mice but significantly decreased in the lung tissues of asthmatic mice treated with QF. Similarly, mice with OVA-induced asthma showed a significantly higher expression of NLRP3, Caspase-1, IL-1β in lung tissues, and QF can significantly downregulate the expression levels of these proteins. These results suggest that the potential mechanism of QF to alleviate airway inflammation in asthma by regulating respiratory microbiota may be related to downregulation of MAPK and NOD-like receptor signaling pathways. However, there were still many limitations to our research. For example, the *in vivo* sample size is relatively small. In addition, more studies are needed to confirm the strong link between QF in reducing airway inflammation and regulating respiratory microbiota.

In conclusion, we observed the anti-airway inflammation effect of QF in OVA-induced asthmatic mice, and for the first time, we explored the effect of QF on the composition of the respiratory microbiota in asthmatic mice. Our study found that QF can regulate the composition of respiratory microbiota in asthmatic mice, and metagenomic function prediction suggests that QF can downregulate MAPK and NOD-like receptor signaling pathways that are significantly upregulated in asthmatic mice. Immunohistochemical results showed that QF could downregulate the expression of p-JNK, p-P38, NLRP3, Caspase-1, and IL-1β, which are the key proteins in the signaling pathways of lung tissue. This study provides a theoretical basis for effective use of QF in asthmatic airway inflammation.

## Data Availability

The data presented in the study are deposited in the NCBI repository, accession number PRJNA730096 and SRP320140.

## Supplementary material

SUPPLEMENTARY FIGURE S1 Total ion chromatography (TIC) of the blank samples. TIC of the blank samples in positive ion mode; **(B)** Total ion chromatography of the blank samples in negative ion mode.

SUPPLEMENTARY FIGURE S2 The leukocyte cell type counts in BALF samples. The BALF was centrifuged and the precipitated cells were counted after staining by Wright-Giemsa. Data are expressed as the mean ± standard deviation (SD) and were performed with one-way ANOVA followed by Tukey’s tests. (*n* = 6, ^△^ *p* ≤ 0.05 vs. the OVA group, ^△△^ *p* ≤ 0.01 vs. the OVA group; ^#^ *p* ≤ 0.05 vs. the budesonide (BUD) group, ^##^ *p* ≤ 0.01 vs. the BUD group; ^◆^ *p* ≤ 0.05 vs. the QFM group, ^◆◆^ *p* ≤ 0.01 vs the QFM group; ns: no difference; line over bars indicates that all groups are include.).
