# Supplementary figures and images for: Anti-inflammatory activities of Qingfei oral liquid and its influence on respiratory microbiota in mice with ovalbumin-induced asthma
Source: Front Pharmacol. 2022 Aug 23;13:911667. doi: 10.3389/fphar.2022.911667 (PMC9445488; doi:10.3389/fphar.2022.911667)

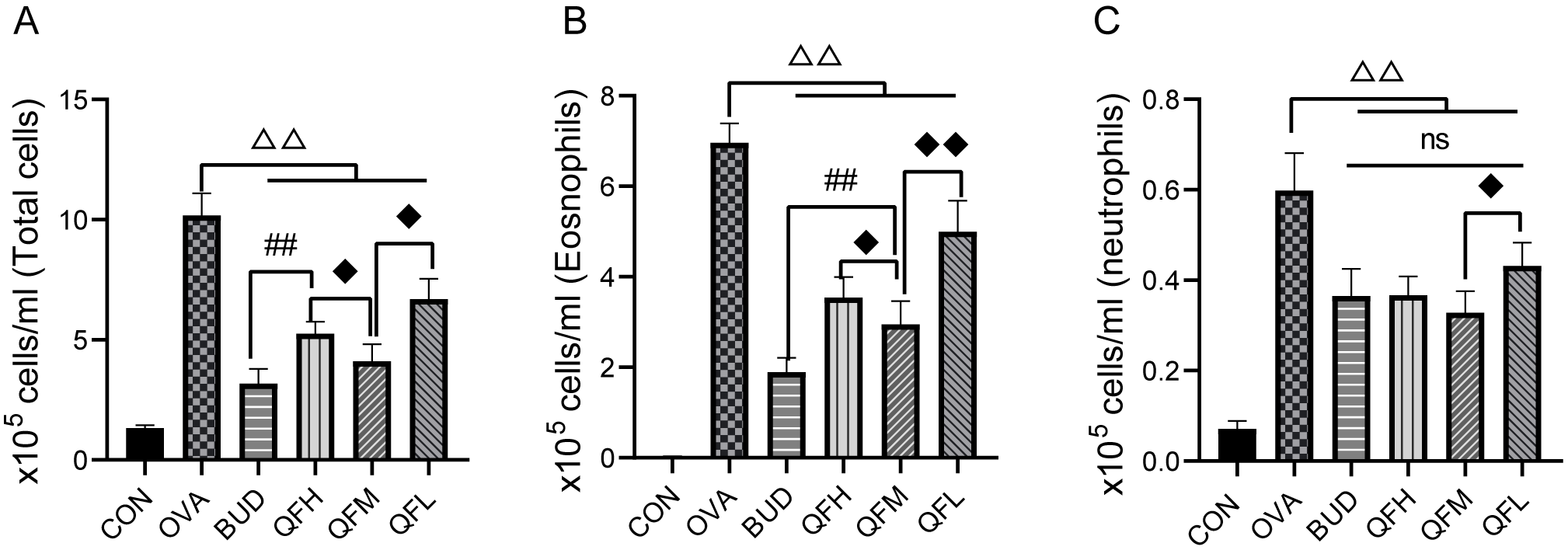

Supplement: Supplementary file 1 [file Image2.tif]

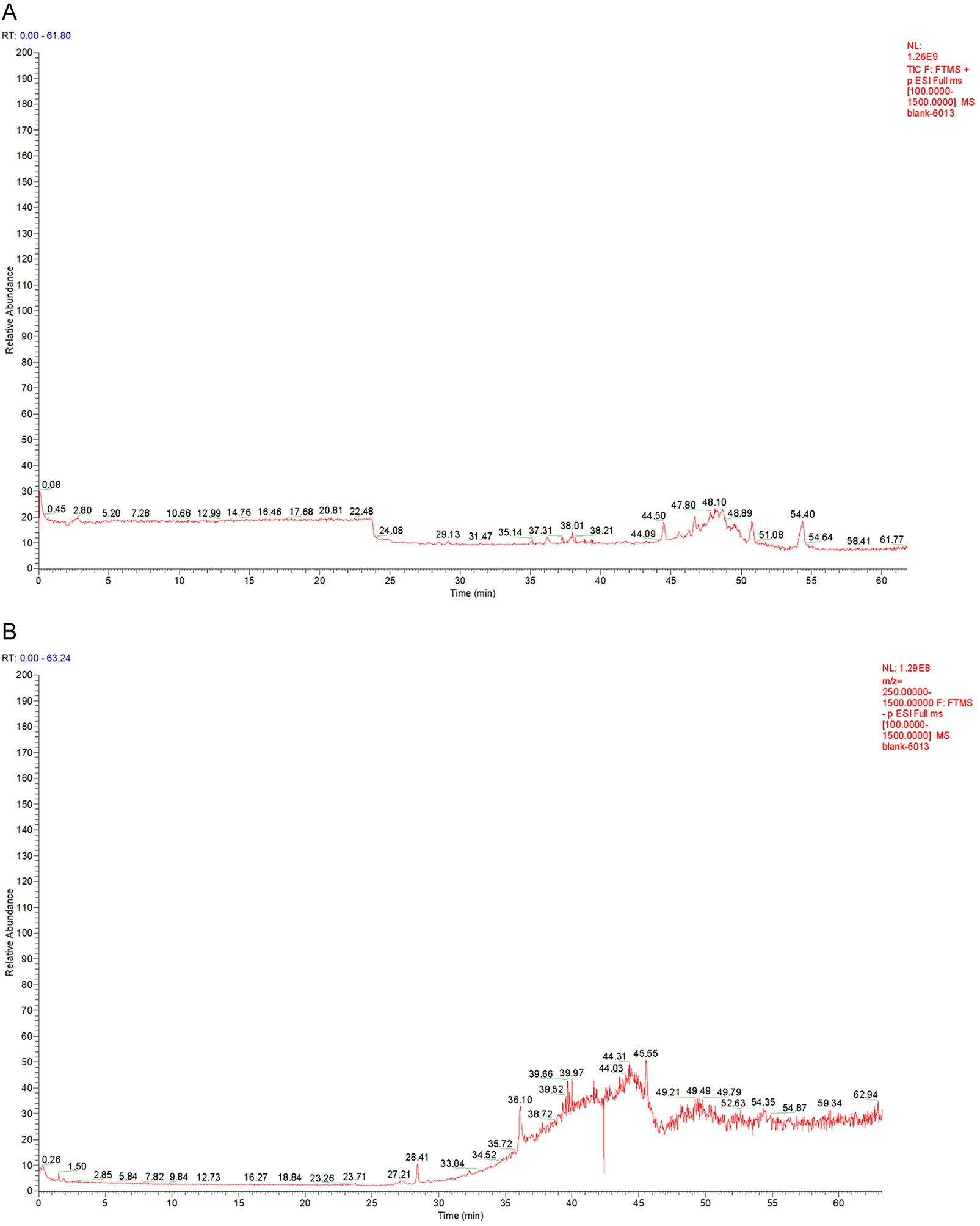

Supplement: Supplementary file 2 [file Image1.tif]
