# Supplementary material for: Anti-inflammatory activities of Qingfei oral liquid and its influence on respiratory microbiota in mice with ovalbumin-induced asthma
Source: Front Pharmacol. 2022 Aug 23;13:911667. doi: 10.3389/fphar.2022.911667 (PMC9445488; doi:10.3389/fphar.2022.911667)
Supplement: Supplementary file 3 [file Table2.DOCX]

Qiime2 analyzes the process code

qiime tools import \

--type 'SampleData[PairedEndSequencesWithQuality]' \

--input-path con-ova-manifest \

--output-path con-ova-demux.qza \

--input-format PairedEndFastqManifestPhred33V2

qiime demux summarize \

--i-data con-ova-demux.qza \

--o-visualization con-ova-demux.qzv

qiime dada2 denoise-paired \

--i-demultiplexed-seqs con-ova-demux.qza \

--p-trim-left-f 19 \

--p-trim-left-r 20 \

--p-trunc-len-f 290 \

--p-trunc-len-r 270 \

--p-n-threads 8 \

--o-table con-ova-table.qza \

--o-representative-sequences con-ova-rep-seqs.qza \

--o-denoising-stats con-ova-denoising-stats.qza

qiime feature-table summarize \

--i-table con-ova-table.qza \

--o-visualization con-ova-table.qzv \

--m-sample-metadata-file con-ova-metadata.tsv

qiime feature-table tabulate-seqs \

--i-data con-ova-rep-seqs.qza \

--o-visualization con-ova-rep-seqs.qzv

qiime metadata tabulate \

--m-input-file con-ova-denoising-stats.qza \

--o-visualization con-ova-denoising-stats.qzv

qiime phylogeny align-to-tree-mafft-fasttree \

--i-sequences con-ova-rep-seqs.qza \

--o-alignment con-ova-aligned-rep-seqs.qza \

--o-masked-alignment con-ova-masked-aligned-rep-seqs.qza \

--o-tree con-ova-unrooted-tree.qza \

--o-rooted-tree con-ova-rooted-tree.qza

qiime diversity core-metrics-phylogenetic \

--i-phylogeny con-ova-rooted-tree.qza \

--i-table con-ova-table.qza \

--p-sampling-depth 6000 \

--m-metadata-file con-ova-metadata.tsv \

--output-dir con-ova-core-metrics-results

qiime diversity alpha-group-significance \

--i-alpha-diversity con-ova-core-metrics-results/faith_pd_vector.qza \

--m-metadata-file con-ova-metadata.tsv \

--o-visualization con-ova-core-metrics-results/faith-pd-group-significance.qzv

qiime diversity alpha-group-significance \

--i-alpha-diversity con-ova-core-metrics-results/evenness_vector.qza \

--m-metadata-file con-ova-metadata.tsv \

--o-visualization con-ova-core-metrics-results/evenness-group-significance.qzv

qiime diversity alpha-group-significance \

--i-alpha-diversity con-ova-core-metrics-results/shannon_vector.qza \

--m-metadata-file con-ova-metadata.tsv \

--o-visualization con-ova-core-metrics-results/shannon-group-significance.qzv

qiime diversity beta-group-significance \

--i-distance-matrix con-ova-core-metrics-results/unweighted_unifrac_distance_matrix.qza \

--m-metadata-file con-ova-metadata.tsv \

--m-metadata-column group \

--o-visualization con-ova-core-metrics-results/unweighted-unifrac-group-significance.qzv \

--p-pairwise

qiime diversity beta-group-significance \

--i-distance-matrix con-ova-core-metrics-results/weighted_unifrac_distance_matrix.qza \

--m-metadata-file con-ova-metadata.tsv \

--m-metadata-column group \

--o-visualization con-ova-core-metrics-results/weighted-unifrac-group-significance.qzv \

--p-pairwise

qiime diversity beta-group-significance \

--i-distance-matrix con-ova-core-metrics-results/bray_curtis_distance_matrix.qza \

--m-metadata-file con-ova-metadata.tsv \

--m-metadata-column group \

--o-visualization con-ova-core-metrics-results/bray-curtis-group-significance.qzv \

--p-pairwise

qiime diversity alpha-rarefaction \

--i-table con-ova-table.qza \

--i-phylogeny con-ova-rooted-tree.qza \

--p-max-depth 10000 \

--m-metadata-file con-ova-metadata.tsv \

--o-visualization alpha-rarefaction.qzv

qiime feature-classifier classify-sklearn \

--i-classifier classifier-v3v4.qza \

--i-reads con-ova-rep-seqs.qza \

--o-classification con-ova-taxonomy.qza

qiime metadata tabulate \

--m-input-file con-ova-taxonomy.qza \

--o-visualization con-ova-taxonomy.qzv

qiime taxa barplot \

--i-table con-ova-table.qza \

--i-taxonomy con-ova-taxonomy.qza \

--m-metadata-file con-ova-metadata.tsv \

--o-visualization con-ova-taxa-bar-plots.qzv

Train the classifie-v3v4

qiime tools import \

--type 'FeatureData[Sequence]' \

--input-path gg_13_8_otus/rep_set/99_otus.fasta \

--output-path 99_otus.qza

qiime tools import \

--type 'FeatureData[Taxonomy]' \

--input-format HeaderlessTSVTaxonomyFormat \

--input-path gg_13_8_otus/taxonomy/99_otu_taxonomy.txt \

--output-path ref-taxonomy.qza

time qiime feature-classifier extract-reads \

--i-sequences 99_otus.qza \

--p-f-primer ACTCCTACGGGAGGCAGCA \

--p-r-primer GGACTACHVGGGTWTCTAAT \

--o-reads ref-seqs.qza

time qiime feature-classifier fit-classifier-naive-bayes \

--i-reference-reads ref-seqs.qza \

--i-reference-taxonomy ref-taxonomy.qza \

--o-classifier classifier_gg_13_8_99_V3-V4.qza

Picrust related files

qiime tools import \

--input-path 13_5_97_otus.fasta \

--output-path ref-sequences.qza \

--type 'FeatureData[Sequence]'

qiime vsearch cluster-features-closed-reference \

--i-sequences con-ova-rep-seqs.qza \

--output-dir closed-ref-otu/ \

--i-table con-ova-table.qza \

--i-reference-sequences ref-sequences.qza \

--p-perc-identity 0.97 \

--p-threads 8

qiime tools export \

--input-path closed-ref-otu/clustered_table.qza \

--output-path closed-ref-otu/

biom convert \

--to-tsv -i closed-ref-otu/feature-table.biom -o closed_reference_otu_table.tsv

Related file download method

wget \

-O "gg_13_8_otus.tar.gz" \

" ftp://greengenes.microbio.me/greengenes_release/gg_13_5/gg_13_8_otus.tar.gz"

wget \

-O "gg_13_5_otus.tar.gz" \

"ftp://greengenes.microbio.me/greengenes_release/gg_13_5/gg_13_5_otus.tar.gz"

wget \

-O "STAMP_2_1_3.exe" \

"https://github.com/dparks1134/STAMP/releases/download/v2.1.3/STAMP_2_1_3.exe"
